# Specific collagen peptides supplementation increases collagen type I content in skeletal muscle after 12 weeks of high-load resistance training: a randomized controlled trial

**DOI:** 10.3389/fphys.2026.1839695

**Published:** 2026-06-15

**Authors:** Simon Jerger, Jakob Lindberg Nielsen, Christoph Centner, Jonas Mathiesen, Charlotte Suetta, Steffen Oesser, Albert Gollhofer, Per Aagaard, Daniel König

**Affiliations:** 1 Department of Sport and Sport Science, University of Freiburg, Freiburg, Germany; 2 Research Unit for Muscle Physiology and Biomechanics, Department of Sports Science and Clinical Biomechanics, University of Southern Denmark, Odense, Denmark; 3 Department of Geriatrics, Central and West Zealand Hospital, Slagelse, Denmark; 4 Geriatric Research Unit, Department of Geriatric and Palliative Medicine, Copenhagen University Hospital Bispebjerg and Frederiksberg, Copenhagen, Denmark; 5 Geriatric Research Unit, Department of Medicine, Copenhagen University Hospital Herlev and Gentofte, Copenhagen, Denmark; 6 Collagen Research Institute, Kiel, Germany; 7 Centre of Sports Science, Department for Nutrition, Exercise and Health, University of Vienna, Vienna, Austria; 8 Department for Nutrition, Exercise and Health, Faculty of Life Sciences, University of Vienna, Vienna, Austria

**Keywords:** collagen, extracellular matrix, intramuscular connective tissue, resistance training, skeletal muscle, specific collagen peptides

## Abstract

**Objective:**

The extracellular matrix is essential for skeletal muscle function, providing structural support and facilitating force transmission from muscle fibers to tendons and the skeletal system. Collagen structures, known for their high plasticity, adapt to various stimuli, including mechanical loading during high-load resistance training. The present study aimed to assess the impact of specific collagen peptide (SCP) supplementation, combined with resistance training, on the levels of intramuscular collagen (COL) types I, III, and IV, and to assess the change in intramuscular fibroblast content.

**Methods:**

In a randomized, placebo-controlled study, 29 healthy male participants completed 12-week high-load resistance training (70-85% of 1 repetition maximum) with daily supplementation of 15g of SCP or placebo (PLA). Before and after the intervention period, muscle biopsies from the vastus lateralis muscle were obtained to quantify intramuscular collagen amount and fibroblast density.

**Results:**

SCP supplementation together with exercise led to increased (p = 0.026) collagen type I content (29.8%) compared to training with placebo (9.9%). Collagen types III and IV content increased significantly across the pooled sample (p < 0.05) with no significant group x time interaction. Fibroblast density did not change over the time course of intervention, either within or between groups.

**Conclusion:**

Endomysial collagen type I content increased in response to resistance training when supplemented with SCP compared to placebo. The observed increase in intramuscular collagen content may contribute to improved structural support of skeletal myofibers.

## 1 Introduction

Collagen is the most abundant structural protein in humans and accounts for one-third of all protein in the human body (Khatri et al., 2021). As a component of the extracellular matrix (ECM), collagens are crucial for the mechanical stability of various tissues, including skeletal muscle (Frantz et al., 2010).

In skeletal muscle, extracellular collagen facilitates force transmission from muscle fibers to the tendons and bones (Gillies and Lieber, 2011; Holwerda and van Loon, 2022), thus playing an important role in the execution of movement. Within skeletal muscle tissue, collagen types I and III are the most abundant of the 28 collagen subtypes (Ricard-Blum, 2011), accounting for approximately 74% of the total collagen content of human skeletal muscle (McKee et al., 2019). The fibril-forming properties of collagen I and III are known to contribute to the tensile strength, stiffness, and elasticity of muscle tissue (Kovanen, 2002; Csapo et al., 2020). In addition, type IV collagen is the main component of the myofiber basement membrane (Kjaer, 2004), where it forms network-forming, interdigitated structures that provide important membrane stability (Kjaer, 2004; Abreu-Velez and Howard, 2012).

Although collagen was previously considered metabolically stable (Bishop and Laurent, 1995), it is now well established that intramuscular connective tissue undergoes continuous remodeling in response to mechanical loading and that resistance exercise is a potent stimulus for collagen turnover (Kjaer, 2004; Babraj et al., 2005; Moore et al., 2005; Mavropalias et al., 2022). Whether nutritional approaches can enhance exercise-induced collagen remodeling remains unclear. While one study reported an increase in intramuscular connective tissue protein synthesis rates during the later phase of post-exercise recovery following whey protein ingestion (Holm et al., 2017), the majority of studies found no additive effect of leucine-rich protein supplementation on exercise-induced collagen synthesis rates (Holm et al., 2009; Trommelen et al., 2020; Aussieker et al., 2023).In contrast, collagen peptides may support ECM remodeling by providing targeted amino acid substrates for collagen synthesis (e.g., glycine, proline, hydroxyproline), and through the potential signaling properties of collagen-derived di- and tripeptides such as prolyl-hydroxyproline (Pro-Hyp) and hydroxyprolyl-glycine (Hyp-Gly), which have been shown to stimulate fibroblast proliferation and ECM production *in vitro* (Ohara et al., 2010; Ide et al., 2021)Notably, collagen-derived supplements have been reported to induce structural and/or functional adaptations in collagen-rich tissues such as bone (Guillerminet et al., 2010; Watanabe-Kamiyama et al., 2010; König et al., 2018), articular cartilage (McAlindon et al., 2011) and tendon (Baar, 2019; Praet et al., 2019; Jerger et al., 2022, 2023; Lee et al., 2023). In their narrative review, Holwerda and van Loon (2022) suggest that collagen products high in glycine and proline may stimulate connective tissue synthesis when administered in parallel with concurrent exercise stimuli. Supporting a stimulatory effect of collagen supplementation on intramuscular connective tissue, a greater acute increase in collagen type I synthesis was observed after gelatine (a collagen derivative) compared with placebo in response to a single session of rope-skipping exercise (Shaw et al., 2017). In contrast, recent experiments (Kirmse et al., 2024) showed that adding collagen supplementation to one week of high-volume resistance-type exercise (combining resistance and plyometric training) did not lead to a greater increase in intramuscular connective tissue protein synthesis, in the quadriceps muscle, compared to intake of non-caloric placebo. Together, these findings highlight that the effects of collagen peptide supplementation on intramuscular connective tissue remodeling including collagen adaption remain inconclusive, particularly with respect to longer-term resistance training interventions in humans. No study to date has systematically examined whether prolonged collagen peptide supplementation during high-load resistance training leads to changes in specific intramuscular collagen subtypes (types I, III, and IV).

Fibroblasts are the primary ECM producing cells in skeletal muscle, and are responsible for the synthesis and maintenance of interstitial collagens, especially collagen types I and III (Chapman et al., 2016), and thus play an important role to muscle fiber structural integrity and ECM remodeling (Chiquet et al., 2009; Plikus et al., 2021). Through mechanotransduction, fibroblasts are highly sensitive to mechanical loadings, enabling adaptive regulation of ECM remodeling in response to exercise (Bohm et al., 2015). As such, fibroblasts may represent a key cellular link through which both mechanical loading and nutritional factors, such as collagen peptide supplementation, influence ECM adaptation in skeletal muscle.

While resistance exercise is one of the most potent stimuli for skeletal muscle ECM remodeling, most research has focused on muscle fiber adaptations, whereas the connective tissue compartment and its primary ECM-producing cells have received comparatively less attention. Data on the effects of protein supplementation in this context are scarce and largely limited to *in vitro* findings in dermal fibroblasts showing that collagen peptides stimulate proliferation and activation, while *in vivo* human studies have not assessed fibroblast-related outcomes (Inacio et al., 2024). Thus, it remains unknown whether long-term high-load resistance training combined with collagen peptide supplementation modulates intramuscular collagen remodeling at the level of specific subtypes (I, III, and IV) and whether such adaptations are associated with changes in intramuscular fibroblast content.

The present study, therefore, investigated the effect of 12 weeks of high-load resistance training (HLRT) combined with daily SCP on endomysial collagen types I, III, and IV compared with HLRT plus placebo. In addition, intramuscular fibroblast content was assessed to provide cellular-level context for potential ECM remodeling processes. To our knowledge, this is the first study to simultaneously examine intramuscular collagen subtype-specific adaptations and fibroblast-related outcomes in response to long-term collagen peptide supplementation during resistance training in humans. We hypothesized that 12 weeks of HLRT combined with SCP supplementation would increase intramuscular collagen content (type I, III, IV) to a greater extent than HLRT and placebo, potentially accompanied by increased fibroblast density.

## 2 Materials & methods

### 2.1 Study design

The present data were obtained as part of a larger ongoing study investigating the effects of a SCP supplementation on general skeletal muscle adaptations induced by resistance training. A randomized, controlled, double-blind study design was employed to examine the effects of daily SCP or placebo supplementation combined with 12 weeks of resistance training on skeletal muscle connective tissue collagen types I, III, and IV content and fibroblast density.

One week before the intervention period, participants completed a preliminary screening involving a full medical history and general medical examination confirm eligibility with the study-specific inclusion criteria (see: “Participants”). Afterwards, participants were randomly assigned to the intervention or control group supplemented with either SCP or placebo, respectively. The order of allocation was determined by a computerized random number generator (Microsoft Excel; Version 365; Microsoft Corporation, Redmond, WA, USA). All outcome assessors, the statistical analysis group, exercise instructors and participants were blinded to group assignments. Before and after the 12-week intervention, a muscle biopsy was taken from the right VL muscle of each participant.

### 2.2 Participants

A total of 30 male participants were included in the study, with an age range of 18 to 29 years and a body mass index (BMI) between 18.5 kg/m^2^ and 25 kg/m^2^. This study was part of a larger research project on the effects of SCP supplementation on skeletal muscle adaptations to resistance training, from which acute gene expression responses to a single bout of HLRT have been reported previously (Centner et al., 2022). The *a priori* sample size calculation was performed for the overarching project based on effect sizes from related functional and molecular outcomes, rather than for the histological endpoints presented here. Prior to the intervention, all participants were found to be moderately active (~150 min/week of moderate or 75 min/week of vigorous exercise [e.g., team sports, cycling]) with no regular participation in strength training during the last 12 months. Participants diagnosed with acute or chronic diseases, cardiac problems, or metabolic disorders were excluded from the study. All study procedures were approved by the local Ethics Committee of the University of Freiburg (study code: 380/19) in accordance with the Declaration of Helsinki and registered in the German Clinical Trials Register (DRKS00027112). All participants were informed about the study procedures and the potential risks of the study before giving their written informed consent.

Dietary intake three days prior to pre and post biopsy samplings was recorded by all participants using a standardized dietary registration protocol (Burd et al., 2010). All participants were instructed to maintain their usual diet until the evening before the experiment, and to refrain from any exercise as well as alcohol and caffeine intake 48 hours prior to arrival at the laboratory (Camera et al., 2015). For the final food intake 12 hours before the experimental trial, participants received a pre-packed meal. The protein content of the final meal was set at 0.4 g per kg body mass. This amount is aligned with current recommendations for trained male athletes (Schoenfeld and Aragon, 2018) and was derived from a targeted total daily protein intake of approximately 1.2–1.4 g per kg body mass (Tang et al., 2009; König et al., 2020). The quantity of calories in the meal was adjusted to meet the estimated total caloric requirement according to the Benedict-Harris equation using a physical activity level (PAL) value of 1.6 (Moore et al., 2009). This combined approach of habitual dietary monitoring, randomization, and short-term pre-biopsy standardization reflects established methodological practice to reduce systematic bias in group comparisons in exercise–nutrition trials where full dietary control is not feasible. Under these conditions, remaining dietary variability is not expected to differentially affect group comparisons and is therefore unlikely to induce systematic bias between groups (Krishnan et al., 2020; Martínez-López et al., 2022).

### 2.3 Supplementation procedures

The interventional supplement consisted of 15 g SCP (BODYBALANCE^®^, Gelita AG, Eberbach, Germany). The placebo consisted of non-caloric silicon dioxide adjusted to a volume-equivalent amount of the active supplement to ensure comparable appearance and mouthfeel, thereby maintaining blinding conditions. A non-caloric, inert placebo was used to isolate the additive effects of collagen peptide supplementation on resistance training–induced adaptations. The exact amino acid composition of the interventional supplement is listed in **Table 1**. All participants were advised to consume the test product dissolved in 250 ml of water directly after exercise on training days, and at the same time of day on non-training days. The daily intake of the supplements was either directly monitored or queried by the trainer and recorded in participants’ training protocol. A minimum adherence rate of 80% (supplement intake on ≥80% of intervention days) was required for inclusion in the final analyses. Both supplements consisted of white, tasteless powders that could not be differentiated by flavor, color or solubility.

**Table 1:** Amino acid composition of the SCP supplement.

Amino acid	Weight [%]	Mol [%]
Hydroxyproline	11.3	9.6
Aspartic Acid	5.8	4.8
Serine	3.2	3.4
Glutamic acid	10.1	7.5
Glycine	22.1	32.3
Histidine	1.2	0.8
Arginine	7.8	5.0
Threonine	1.8	1.7
Alanine	8.5	10.5
Proline	12.3	11.8
Tyrosine	0.9	0.5
Hydroxylysine	1.7	1.2
Valine	2.4	2.3
Methionine	0.9	0.9
Lysine	3.8	2.9
Isoleucine	1.3	1.1
Leucine	2.7	2.3
Phenylalanine	2.1	1.4

SCP, specific collagen peptides.

### 2.4 Training protocol

The training intervention consisted of 12 weeks of 3 weekly exercise sessions. Successive exercise sessions were separated by at least 36 hours of rest to allow adequate recovery. Each exercise session was preceded by a 10-minute warm-up on a stationary cycle ergometer (50–80 Watts). The training protocol included three sets of knee extension and leg press exercises, respectively. In week 1-2, an exercise load of 70% of 1 repetition maximum (1RM) was employed, with loads progressing to 75, 80 and 85%-1RM in weeks 3-4, 5–8 and 9-12, respectively. At the same time points, the number of repetitions decreased from 15 to 12, 10 and 8 repetitions per set. Individual 1RM tests were performed before the intervention and after week 2, 4 and 8 to ensure progressive load increments throughout the study. Standardized resting periods were one minute between sets and three minutes between exercises. Leg press and knee extension were performed from 90° knee angle to nearly full extension. In addition, upper body exercises in the form of latissimus pull, supine bench press, and sit-ups were included in the exercise protocol with the aim of to increasing exercise compliance. A minimum training adherence of 80% was required for inclusion, defined as the full completion of ≥ 29 of the prescribed training sessions.

### 2.5 Muscle tissue sampling and handling

One week before and within one week after completing the intervention period, all participants arrived at the laboratory between 7.00 and 8.00 a.m. after an overnight fast (Hulmi et al., 2009). After a 30-min resting period, a muscle biopsy was obtained from the VL muscle of the right leg. For this purpose, a 5-mm Bergström needle with manual aspiration was used after local anesthesia (2% xylocitin) (Nielsen et al., 2012; Suetta et al., 2013). The tissue sample was cleaned of excess blood using blotting paper. Afterwards, the sample was frozen in isopentane pre-cooled in liquid nitrogen. All samples were stored at -80 °C for later analysis (Nielsen et al., 2012; Suetta et al., 2013).

### 2.6 Immunofluorescence analysis

In brief, transverse cut serial sections (8 µm) of the muscle tissue biopsies were fixed (4% formaldehyde, Triton-X buffer) for 10 min, washed in PBS (3x3 min), and subsequently blocked (X0909, Dako, Glostrup, Denmark) for 10 min (Nielsen et al., 2012; Suetta et al., 2013). A staining was performed to visualize collagen I (Sigma-Aldrich (SA), C2456) and IV (SA, MAB3303). A second analysis was performed to visualize collagen III (SA, C7805) and fibroblasts (anti-transcription factor 4 (TCF‐4) recognizing transcription factor 7‐like 2 (TCF7L2, Cell Signalling, 2569) (Mackey et al., 2017). The following secondary antibodies were used (order listed: Goat anti-Rabbit (SA, A21424) Goat Anti-mouse 488 (SA, A21121), biotinylated anti-rabbit (Vector)/DyLight-549 (Vector, SA-5549). Sections were initially incubated with primary antibodies for 60 minutes at room temperature, followed by a wash step (PBS, 3x3 min), while subsequently incubated with secondary antibodies for 60 minutes. Finally, sections were washed in PBS, stained with 4’,6-diamidino-2-phenylindole (DAPI) and mounted in medium (Vector, H-5700) and stored protected from light at 5 °C. Sections in which primary antibodies were omitted served as negative controls.

Stained sections were visualized using fluorescent microscopy (Axio Imager M1, Carl Zeiss, Germany) and a high-resolution AxioCam (Carl Zeiss). Images were obtained using standardized settings (cf. x10, exposure). All analyses were performed manually in AxioVision image analysis software (AxioVision 4.6, Carl Zeiss). Endomysial collagen flourescence density was quantified as mean signal intensity per area using a standardized grid of circular regions of interest (ROIs, radius 6 µm). For each myofiber, only the most centrally positioned ROI was kept and systematically translated such that its center aligned with the sarcolemmal region, thereby ensuring consistent endomysial sampling and minimizing selection bias across images. Any immunoreactive areas were excluded only if predefined criteria were met, including presence of imaging artifacts (e.g., tissue folds) or inclusion of non-representative structures (e.g., vessels) with disproportionally high signal intensity. These criteria were applied uniformly across all images using identical analysis settings. All density measures were background corrected and expressed in arbitrary units. Fibroblasts were identified as TCF7L2-positive nuclei, defined by co-localization of TCF7L2 immunostaining with DAPI (TCF7L2^+^/DAPI^+^). Only TCF7L2^+^-nuclei with clear spatial localization within the endomysium were classified as fibroblasts, in accordance with established criteria (Mackey et al., 2017). Fibroblast abundance was quantified and expressed relative to the number of analyzed myofibers. An average of 319 ± 139, 188 ± 92 and 247 ± 129 myofibers were analyzed per muscle biopsy for collagen I/IV, collagen III, and fibroblasts, respectively. A minimum of 50 analyzed myofibers was set for analyses to be representative, resulting in two participants being withdrawn from the fibroblast analysis (final analyses involved SCP/PLA: 14/13). All analyses, including ROI placement and signal quantification, were performed by an investigator blinded to participant ID, group allocation, and time point.

### 2.7 Lifestyle parameters

The self-reported physical activity performed beyond the intervention program was assessed using the Freiburg Questionnaire of Physical Activity (Frey et al., 1999) before the beginning of the intervention.

Additionally, dietary intake was assessed using three-day dietary records (two weekdays and one weekend day) collected from all participants during the week before and after the intervention period. From this, macronutrient and total energy intake were analyzed using Nutriguide 4.6 [Nutri Science GmbH; Hausach, Germany]. To exclusively assess the impact of the intervention, all participants were instructed to maintain their habitual diet and habitual physical activity (aside from the standardized training intervention) throughout the entire study period.

### 2.8 Statistical analysis

All statistical analyses were performed blinded to group allocation and participant identity. Statistical analyses were conducted using SPSS [version 27.0, IBM]. All results are presented as group mean ± SD if not otherwise indicated. Since all variables showed normal distribution according to Shapiro-Wilk testing, parametric (i.e. Gaussian) tests were used for statistical analysis. Baseline group differences were assessed using unpaired t-tests. After confirming homogeneity of variances by Levene’s test, repeated measurement ANOVA (rmANOVA) was used to assess group x time interaction effects. Paired t-tests were applied for within-group comparisons. The level of significance was set to p < 0.05. Effect sizes are presented by the partial eta squared (ƞp²) with ƞp² > 0.01 indicating small, ƞp² > 0.06 indicating medium and ƞp² > 0.14 indicating large effect sizes (Cohen, 1988), and are reported with 95% confidence intervals.

## 3 Results

### 3.1 Participant characteristics

In total, n = 29 of the n = 30 participants completed all study procedures, with n = 14 in the SCP group and n = 15 in the PLA group. One participant dropped out due to relocation during the intervention period. **Table 2** shows the anthropometric characteristics at baseline for all participants included in the analysis.

**Table 2:** Baseline anthropometric characteristics.

Variable	SCP	PLA
Age (years)	25.4 ± 2.4	24.0 ± 2.6
Height (cm)	177.9 ± 8.1	178.5 ± 7.3
Weight (kg)	72.5 ± 9.9	71.2 ± 7.8
BMI (kg/m²)	22.9 ± 2.2	22.4 ± 2.4

SCP, specific collagen peptides; PLA, placebo; BMI, body mass index. Data are [mean ± SD] (n = 29).

The groups were similar at baseline with respect to age, height, weight and BMI (p > 0.05). There were no significant between-group differences in exercise or supplementation compliance (p > 0.05). All included participants completed 100% (36/36) of the prescribed training sessions and mean self-reported supplement compliance was 99.3 ± 1.4% in SCP and 98.7 ± 1.4% in PLA.

### 3.2 Collagen content

**Figure 1:**
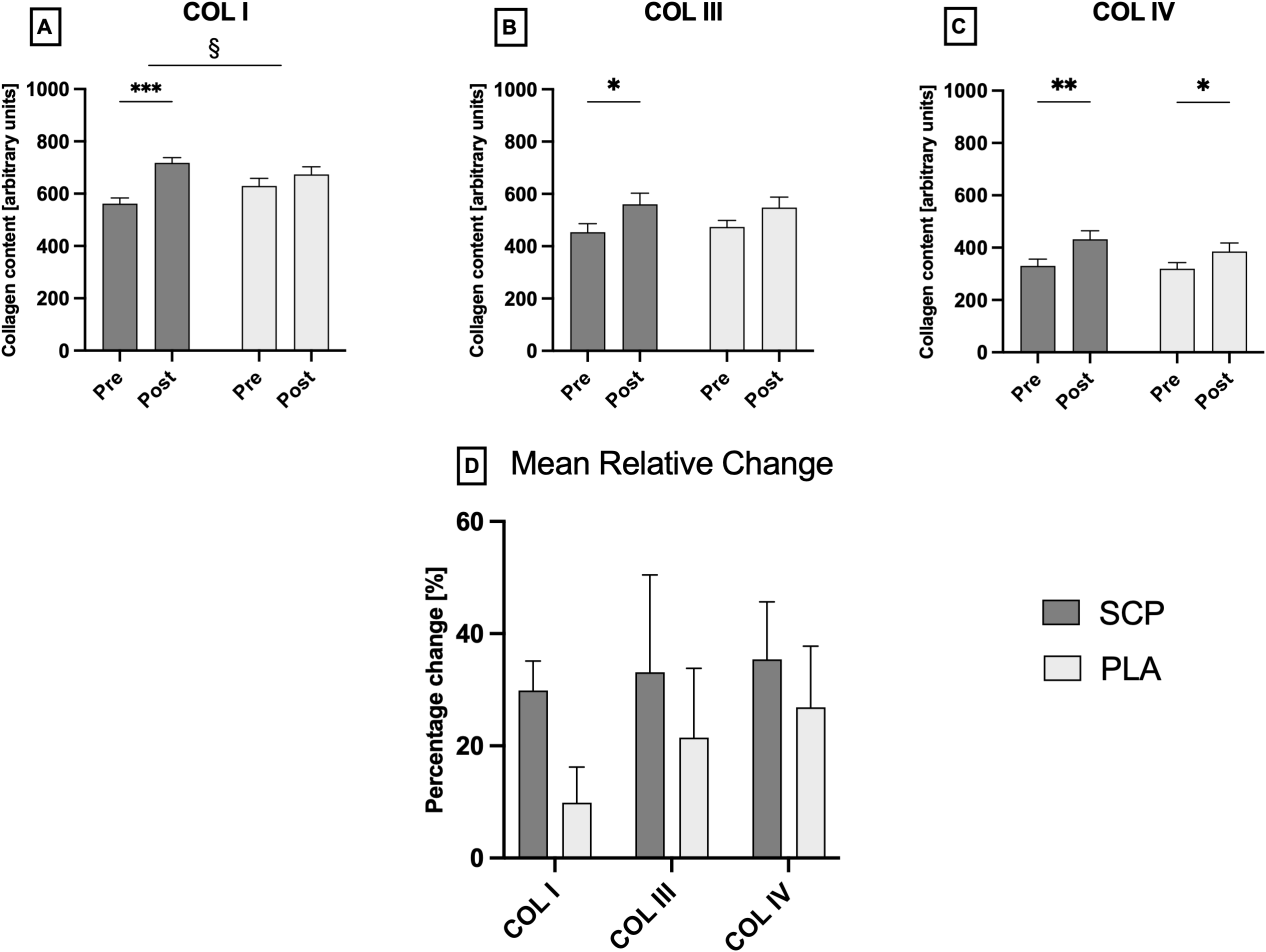
Mean values (± SEM) of intramuscular content of collagen types I, III and IV **(A–C)** and of relative individual changes **(D)** before (Pre) and after (Post) 12 weeks HLRT intervention combined with SCP (dark gray) or PLA supplementation (light gray) with rmANOVA time*group interaction: § p < 0.05 and paired t-tests: *p < 0.05; **p < 0.01 and ***p < 0.001.

For collagen type I, significantly greater increases were observed in SCP (+ 29.8%) compared with PLA (+ 9.9%), as indicated by a significant group × time interaction (p = 0.003) and a large effect size (hp2 = 0.170, 95 % CI [0.05, 0.31]) (**Figure 1A**). Group × time interactions for collagen type III and IV were not statistically significant (p = 0.648 and p = 0.393, respectively), indicating no evidence of differential changes between groups over time for these outcomes. The estimated effect size of the between-group differences in change was small for collagen type IV [ηp² = 0.027, 95% CI (0.00, 0.11)] and trivial for collagen type III [ηp² = 0.008, 95% CI (0.00, 0.06)]. A significant main effect of time was observed for collagen type I (p < 0.001; ηp² = 0.397), collagen type III (p = 0.016; ηp² = 0.195), and collagen type IV (p < 0.001; ηp² = 0.385). No significant baseline differences were observed between groups for any outcome measure (all p > 0.05). Within-group pre–post comparisons indicated increases are reported for descriptive purposes only, and are not part of the inferential analysis of the treatment effects. These analyses show increases in SCPfor all three collagen types (COL I: p < 0.001; COL III: p = 0.028; COL IV: p = 0.001; **Figures 1A–C**), whereas in PLA, collagen content increased for COL IV and remained unchanged for COL I (p = 0.137) and COL III (p = 0.077). These within-condition analyses are reported solely to illustrate the direction of changes within each condition, and to complement the interpretation of the group × time interaction effects, which constitute the basis for between-group inference.

### 3.3 Fibroblast density

**Figure 2:**
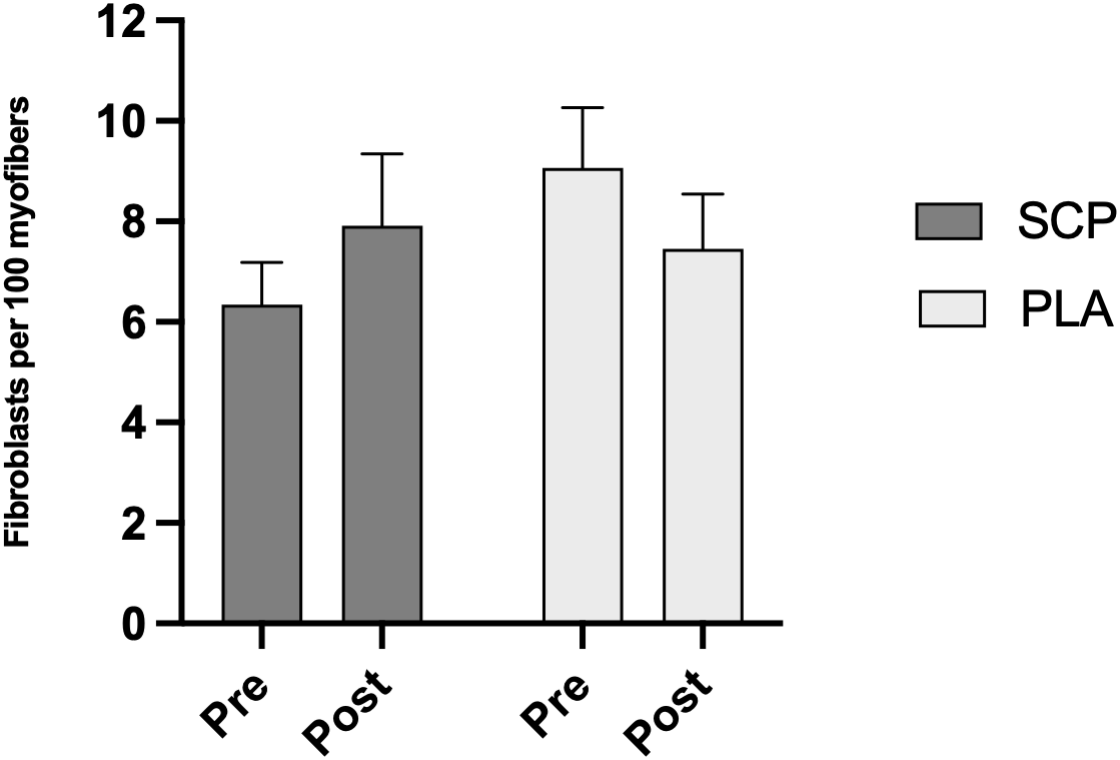
Fibroblast density (no. cells per 100 myofibers) in SCP (dark gray; left; n=14) and PLA (light gray; right; n=13) before (pre) and after (post) 12-wk HLRT intervention (group mean ± SEM).

For fibroblast density, there was no significant group × time interaction (p = 0.124, ηp² = 0.092) and no main effect of time (p = 0.706, ηp² = 0.006). In addition, no changes from pre- to post-measurements were observed in SCP (+24.6%, p = 0.177) and PLA (-17.8%, p = 0.121; **Figure 2**).

### 3.4 Dietary intake and physical activity

Before start of the intervention, no between-group differences were observed in self-reported physical activity–related energy expenditure (SCP: 538 ± 421 kcal/day; PLA: 382 ± 183 kcal/day; unpaired t-test p > 0.05). Likewise, average daily total energy intake and macronutrient distribution did not differ between groups at baseline (p > 0.05) and remained comparable over the course of the intervention, with no significant time x group interaction for any of the examined parameters (p > 0.05; **Table 3**).

**Table 3:** Dietary intake.

Variable		SCP	PLA
Caloric intake [kcal/day]	Pre	2283 ± 521	2208 ± 648
	Post	2416 ± 681	2487 ± 856
Protein [g/day]	Pre	85.2 ± 20.5	88.8 ± 29.5
	Post	87.7 ± 25.6	91.7 ± 29.0
Fat [g/day]	Pre	80.3 ± 30.1	78.6 ± 34.1
	Post	88.6 ± 34.6	83.3 ± 31.8
Carbohydrate [g/day]	Pre	282 ± 71	259 ± 103
	Post	288 ± 103	318 ± 137

SCP, collagen peptides; PLA, placebo. Data are [Mean ± SD] (*n* = 29).

## 4 Discussion

The objective of the present double-blind randomized controlled trial was to determine whether supplementation with SCP during resistance training increases collagen content in intramuscular connective tissue in healthy, moderately active men. The present findings report for the first time an increase in intramuscular type I collagen content after 12 weeks of resistance training and concomitant daily supplementation of 15g of specific collagen peptides compared to the same training regimen combined with placebo.

### 4.1 Collagen content

The SCP group showed a ~3.5-fold greater increase in collagen type I content in comparison to HLRT combined with placebo. This effect was supported by a significant group x time interaction and a large effect size. Thus, the present results indicate that ingestion of SCP during resistance exercise is associated with a greater increase in intramuscular collagen type I accretion of healthy male individuals compared by HLRT and placebo.

Collagen types III and IV did not show statistically significant group × time interactions, indicating that no additional effect of SCP supplementation beyond training alone was observed for these outcomes. A significant main effect of time was present for all collagen types, reflecting an overall increase during the intervention period in both groups. These findings suggest that 12 weeks of HLRT, irrespective of supplementation, were sufficient to elicit measurable changes in the intramuscular collagen content, while no additive effect of SCP could be demonstrated for COL III and IV. Given the limited sample size, small effects cannot be entirely ruled out, and adequately powered studies will be needed to clarify whether nutritional strategies can meaningfully modulate intramuscular collagen remodeling beyond the stimulus provided by resistance training alone.

Recently, it has been discussed whether protein supplementation has an additive effect on exercise-related adaptations of intramuscular connective tissue. Holm et al. (2017) reported a time-dependent effect of ingestion of 18 g whey protein on intramuscular collagen synthesis, with higher fractional synthesis rates observed for whey protein compared with carbohydrate during the late (3–5 h) post-exercise recovery period, whereas no overall effect across the entire recovery period was detected (Holm et al., 2017). However, although amino acids from dietary proteins seems to be incorporated in newly synthesized intramuscular connective tissue proteins, reflecting *de novo* collagen synthesis (Trommelen et al., 2020) most studies have not observed an additive effect of high-quality protein supplementation (whey, casein) either with or without exercise (Babraj et al., 2005; Holm et al., 2009; Trommelen et al., 2020).

Interestingly, a recent study that supplemented participants with hydrolyzed collagen peptides failed to demonstrate an effect on intramuscular connective tissue protein synthesis in response to a one-week training intervention consisting of ~70% of 1RM RT, drop jumping, and rope skipping (Kirmse et al., 2024). Despite significantly elevated concentrations of circulating glycine, proline, and hydroxyproline in the collagen group, no differences in connective or myofibrillar protein synthesis were observed compared to placebo. These divergent outcomes may be explained by differences in training duration and exercise stimuli, respectively. The present study involved 12 weeks of resistance training involving leg press, knee extension at intensities of up to 85% of 1RM. Within tendon research, it is well established that structural adaptations in collagen-rich connective tissues require prolonged intervention durations, combined with sustained high mechanical loading and extended time under tension (Kjaer, 2004; Bohm et al., 2015). Similarly, the training stimulus employed in the present study may have been more effective in inducing the adaptive processes underlying increased intramuscular type I collagen synthesis than a shorter training period (one week) and a protocol imposing different mechanical demands due to the inclusion of plyometric exercises (e.g., rope skipping, drop jumps).

Based on the present observations, collagen peptides may support increases in intramuscular collagen type I accretion when combined with resistance exercise. This effect may be attributable to the specific amino acid profile of collagen peptides (**Table 1**), rich in amino acids (i.e., glycine and proline) with importance for collagen synthesis. Notably, previous metabolic flux analysis revealed that endogenous glycine synthesis is stoichiometrically limited and the glycine content of a typical dietary intake is often insufficient to meet the total daily requirement for metabolic processes, including diurnal collagen production (~10 g per day for a 70 kg adult) (Meléndez-Hevia et al., 2009). With only ~3 g/day endogenously synthesized and 1.5–3 g/day typically consumed via the diet (Meléndez-Hevia et al., 2009), the resulting glycine deficit has been proposed as a potential limiting factor for collagen synthesis, especially under conditions of increased demand associated with i.e. resistance training. It is possible that supplementation with collagen peptides may help bridge this potential gap and promote more effective intramuscular connective tissue remodeling during periods of HLRT in line with recent suggestions (Holwerda and van Loon, 2022). Beyond substrate provision, collagen-derived peptides have been shown in previous studies to act as bioactive signaling molecules by activating receptor-mediated pathways-integrin-, DDR-, and TGF-β-dependent signaling cascades- that regulate collagen synthesis and extracellular matrix remodeling. These pathways have been implicated in the regulation of collagen synthesis and extracellular matrix remodeling, and are considered to be relevant under conditions of increased mechanical loading (Elango et al., 2019; Liu et al., 2019; Elango et al., 2022; Dierckx et al., 2024; Zhang et al., 2025). In the same cohort as the present study, Centner et al. (2022) reported an up−regulation of the MAPK and PI3K−Akt pathways (Centner et al., 2022), which in other experimental contexts have been associated with the regulation of collagen synthesis (Kimoto et al., 2004; Yokoyama et al., 2012).

Notably, Kirmse et al. (2019) reported a greater increase in fat-free mass after 12 weeks of resistance training in young, recreationally active men who received collagen peptides compared with those receiving placebo. The authors attributed part of this effect to an increase in intramuscular connective tissue (Kirmse et al., 2019). Although this hypothesis cannot be verified directly, it is indirectly supported by the present findings of increased collagen type I density. Although collagen represents only a small fraction of total skeletal muscle protein (Holwerda et al., 2026), supplementation may nonetheless enhance the extracellular matrix at a structural level and thereby contribute, at least in part, to the greater gains in fat-free mass previously observed with collagen peptides compared with placebo (Zdzieblik et al., 2015; Jendricke et al., 2019; Oertzen-Hagemann et al., 2019; Zdzieblik et al., 2021).

Given the established role of intramuscular connective tissue in the mechanical behavior of skeletal muscle, such changes may be relevant to tissue-level properties involved in force transmission and load distribution (Purslow, 2020; Finni et al., 2023). However, as the present analysis focused on structural outcome parameters, no conclusions can be drawn regarding effects on functional outcomes such as muscle performance, strength, or neuromuscular function. Accordingly, functional implications remain theoretical and require confirmation in future studies incorporating evaluation of physical performance or mechanical muscle function.

### 4.2 Fibroblast density

The present analysis of intracellular fibroblasts content in the knee extensor muscle (VL) did not show any response to resistance training or additional supplementation (SCP or Placebo). Given that fibroblasts are the primary cellular source of extracellular matrix proteins, including (pro)collagen (Chapman et al., 2016; Purslow, 2020), an increase in collagen accretion could potentially require changes in fibroblast abundance. In the present study, increased intramuscular collagen content occurred over the course of the intervention in the absence of detectable changes in fibroblast density. This suggests that collagen accretion can occur without parallel alterations in fibroblast content under the applied conditions. These data are consistent with prior studies indicating that SCP supplementation may enhance the activity of existing fibroblasts and promote ECM remodeling (Oertzen-Hagemann et al., 2019; Inacio et al., 2024). To date, it is largely unknown to what extent different exercise modalities influence fibroblast content in human skeletal muscle tissue. There is evidence that HLRT increases circulating levels of fibroblast growth factors (Haghighi et al., 2022), which are known to play a role in fibroblast cell proliferation (Yun et al., 2010).

Notably, a specific exercise protocol designed to induce focalized myofiber injury resulted in a four-fold increase in the number of fibroblasts in human VL muscle, with fibroblasts preferentially localized in the proximity of regenerating muscle fibers (Mackey et al., 2017) while accompanied by a 2-fold increase in collagen gene expression (Mackey et al., 2016). In contrast, although strenuous, the present resistance training protocol did not appear to provide sufficient mechanical or regenerative stimuli to increase fibroblast content, suggesting that stronger collagen protein synthesis stimuli and/or more pronounced muscle regeneration may be required to stimulate intramuscular fibroblast proliferation.

### 4.3 Methodological considerations

The trial has some limitations that should be considered when interpreting the findings. The sample size was determined as part of pre-specified randomized controlled trial investigating skeletal muscle adaptations to resistance training combined with collagen peptide supplementation, and was therefore not specifically powered for the present histological outcomes. Accordingly, the relatively small sample size may have limited the ability to detect small, albeit physiological relevant within- and between-group differences in intramuscular outcomes. Future studies, specifically powered for the outcomes of interest, are needed to determine whether supplementation affects ECM properties and the tissue microenvironment, including expression of interstitial collagens and fibroblast content.

Immunofluorescence intensity analysis is inherently semi-quantitative and may be influenced by imaging conditions and ROI selection. Although acquisition settings were kept constant across samples, and ROIs were defined using consistent criteria, a degree of observer-dependent bias cannot be fully excluded.

The present findings should be interpreted within the context of a cohort of young, healthy, recreationally active males without recent resistance training experience. Accordingly, extension of these findings to trained individuals, females, older adults, or clinical populations represents relevant topics for future research.

It is important to note that collagen peptide preparations can vary in composition and pharmacological effects (Schadow et al., 2017), meaning the efficacy of one formulation cannot be generalized to others. Therefore, the results of this study are specific to the collagen peptide product tested and cannot necessarily be extrapolated to other formulations.

## 5 Conclusion

The findings of the present study demonstrated an increased formation of type I collagen in intramuscular connective tissue in response to 12 weeks of HLRT when combined with daily supplementation of 15 grams of bioactive collagen peptides and compared to placebo. Additionally, collagen types III and IV increased significantly from pre to post in SCP, while only collagen type IV increased in PLA, with no significant between-group differences. No changes in intramuscular fibroblast content were observed following training.

## Data Availability

The raw data supporting the conclusions of this article will be made available by the authors, without undue reservation.
